# Immune-inflammatory and metabolic signatures for osteoporosis risk stratification in primary Sjögren’s syndrome: development and internal validation of an interpretable machine-learning model

**DOI:** 10.3389/fimmu.2026.1812285

**Published:** 2026-07-10

**Authors:** Jingqi Dong, Jinle Zhang, Zelin Wang, Shufen Liang

**Affiliations:** Department of Laboratory, The Second Hospital of Shanxi Medical University, Taiyuan, Shanxi, China

**Keywords:** machine learning, osteoporosis, prediction model, primary Sjögren’s syndrome, risk factors, SHAP analysis

## Abstract

**Background:**

Primary Sjögren’s syndrome (pSS) is a systemic autoimmune disease. Osteoporosis (OP) is a common complication in patients with pSS (pSS-OP), which significantly affects their quality of life and prognosis. Currently, there is a lack of efficient and objective clinical tools for the early identification of pSS patients at high risk of osteoporosis, limiting the implementation of precise interventions.

**Objective:**

This study aims to integrate clinical indicators and immunological characteristics to develop and validate a machine learning model for predicting the risk of osteoporosis in patients with pSS, thereby facilitating early clinical identification and decision-making.

**Methods:**

Clinical data were collected from 384 patients with pSS. Lymphocyte subsets and serum cytokine levels of IL-2, IL-4, and IL-6 were measured in all participants. Missing data were handled using multiple imputation, followed by intergroup comparisons. Feature selection was performed using Lasso regression, random forest, and stepwise regression, and the intersection of these methods was used to identify the final predictors. Based on the selected features, nine machine learning models were constructed and compared, including decision tree, k-nearest neighbor, logistic regression, elastic net, random forest, support vector machine, multilayer perceptron, LightGBM, and XGBoost. Model performance was evaluated using ROC curves, calibration curves, decision curve, accuracy, F1 score, sensitivity, specificity, and other metrics. The SHAP method was applied to interpret the optimal model.

**Results:**

Patients were divided into a pSS with OP and a pSS without OP group. Six core predictors were identified: Age, ESR, Urea, MON, Ca, and Fibrinogen. Among the nine models, the XGBoost model demonstrated the best performance. In the training set, the AUC was 0.890, and the F1 score was 0.90. In the test set, the AUC was 0.808, and the F1 score was 0.81. SHAP analysis revealed the following order of feature importance: Age, MON, ESR, Ca, Urea, and Fibrinogen. Among these, Ca contributed negatively to the model prediction, while the remaining features contributed positively.

**Conclusion:**

This study developed and internally validated an XGBoost machine learning model based on clinical and laboratory indicators for predicting osteoporosis risk in patients with pSS. The model demonstrated good discrimination and may assist early risk assessment.

## 1 Introduction

Primary Sjögren’s syndrome (pSS) is a chronic systemic autoimmune disease characterized by the invasion of exocrine glands, such as the lacrimal and salivary glands, and clinically manifests as xerostomia and xerophthalmia (1). The pathogenesis of pSS is complex and involves multiple factors, including genetic predisposition, environmental triggers, and intrinsic defects of the exocrine glands. Both innate and adaptive immunity play critical roles in the development of pSS. The interaction between genetic and environmental factors leads to the activation of immune cells and subsequent autoimmune attacks on epithelial cells of the exocrine glands. Damaged epithelial cells produce chemokines and proinflammatory cytokines, which amplify the autoimmune response through immune infiltration. This cascade results in sustained damage to the exocrine glands and eventually leads to sicca symptoms (2, 3). However, the impact of pSS extends beyond glandular involvement and is frequently accompanied by various systemic complications. Among these, skeletal system involvement represents an important but often overlooked component (4).

Osteoporosis (OP) is a metabolic bone disorder characterized by reduced bone mass and deterioration of bone microarchitecture, leading to increased bone fragility and fracture risk, which seriously impairs quality of life and long-term health outcomes (5). Traditionally, osteoporosis has been viewed as a degenerative disease associated with aging and hormonal changes, whereas autoimmune diseases are primarily characterized by immune-mediated attack on self-tissues. However, accumulating evidence suggests a profound interplay between these two conditions at the pathophysiological level. Studies have demonstrated that patients with various autoimmune diseases frequently exhibit early bone loss and an elevated fracture risk (6, 7). These phenomena cannot be fully explained by reduced physical activity due to disease symptoms or glucocorticoid use; rather, they arise from complex, multi-system, and multi-pathway interactions. First, abnormal vitamin D metabolism constitutes a core link. Vitamin D not only regulates bone metabolism by modulating calcium-phosphate homeostasis (8, 9) but also acts as a key immunomodulator. Its deficiency can exacerbate immune-inflammatory states and promote bone resorption (10, 11). Second, sex hormone imbalance serves as another important hub. Declines in estrogen and androgen levels contribute to lymphocyte infiltration and apoptosis of exocrine gland cells in autoimmune diseases (12, 13), while simultaneously reducing osteoblast activity and diminishing inhibition of osteoclasts (14, 15). These effects collectively drive both glandular dysfunction and bone loss. Third, the RANKL/RANK/OPG axis represents a central pathway in osteoimmunology. Inflammatory cytokines such as IL-6 and TNF-α, produced by activated Th17 cells and other immune cells, significantly upregulate RANKL expression and inhibit OPG (16), thereby strongly promoting osteoclast differentiation and bone resorption (17, 18). In summary, autoimmune diseases and osteoporosis may form a vicious cycle involving immune dysregulation, endocrine disturbance, and bone metabolic imbalance through shared pathways including vitamin D, sex hormones, and the RANKL/RANK/OPG axis.

Patients with pSS are at elevated risk for osteoporosis. Studies report that the prevalence of osteoporosis in pSS patients ranges from 18.5% to 39.5% (19). Multiple clinical observations and meta-analyses have confirmed that, compared with healthy controls, pSS patients exhibit significantly lower bone mineral density (BMD) and a markedly increased risk of osteoporosis and fractures (20, 21). Although the interplay between osteoporosis and autoimmune diseases has been elucidated in conditions such as rheumatoid arthritis and ankylosing spondylitis—where related osteoimmune pathways have become important therapeutic targets—a significant research gap remains in pSS, a common autoimmune disease. pSS is a systemic autoimmune disease characterized by chronic immune activation and B and T cell dysregulation. Increasing evidence suggests that immune imbalance is also involved in bone metabolism, because activated lymphocyte subsets and their related cytokines can influence osteoclast differentiation and bone resorption through the osteoimmune network. Therefore, osteoporosis in pSS may not simply be a coincidental comorbidity, but may be related, at least in part, to the underlying immune abnormalities of pSS. Current understanding of pSS largely focuses on exocrine gland injury and systemic inflammation. The direct impact of the disease itself on bone health, and how its unique pattern of immune cell infiltration and cytokine milieu disrupts bone metabolic homeostasis, still lack systematic investigation. Addressing this gap is essential for comprehensively understanding the systemic burden of pSS and for implementing early bone protection strategies.

Commonly used BMD measurements include dual-energy X-ray absorptiometry (DXA) and quantitative computed tomography (QCT). However, limited availability and low screening rates of DXA constrain its feasibility as a universal osteoporosis screening tool (22), while QCT is less widely adopted in clinical practice due to its higher radiation dose, cost, and technical demands. Therefore, establishing a specific risk prediction model for osteoporosis in pSS is of great clinical importance for early identification of high-risk patients, enabling effective interventions to prevent fractures, and improving long-term prognosis. Machine learning algorithms have shown substantial promise in predictive modeling for various diseases (23), including risk stratification in pSS and some of its complications (24). However, research applying machine learning to predict osteoporosis secondary to pSS remains in its infancy, underscoring the need to develop an effective prediction tool. To address the research gap concerning the mechanistic association and risk prediction between pSS and osteoporosis, this study conducts a retrospective analysis. Machine learning techniques are employed to select key features and construct a multivariable prediction model for identifying pSS individuals at high risk of osteoporosis. Model interpretation is performed using SHapley Additive exPlanations (SHAP) analysis, aiming to provide novel strategies for early diagnosis and intervention.

## 2 Methods

### 2.1 Clinical data

In this study, 402 patients with pSS were enrolled from the Department of Rheumatology at the Second Hospital of Shanxi Medical University between July 2023 and December 2025. To ensure the rigor of case selection, strict inclusion and exclusion criteria were applied to develop a high-quality predictive model for pSS-OP. The inclusion criteria required compliance with either the 2002 American–European Consensus Group criteria or the 2016 American College of Rheumatology/European League Against Rheumatism classification criteria for pSS (25, 26). The exclusion criteria were as follows: presence of other autoimmune diseases; juvenile-onset pSS; concurrent tuberculosis, malignant tumor, or mental illness; continuous use of glucocorticoids for more than two years; and patients with severely missing clinical data. Osteoporosis was diagnosed according to the 2018 Chinese Guidelines for the Diagnosis and Treatment of Senile Osteoporosis. In the present study, the diagnosis was based on DXA, and patients with a T-score ≤ −2.5 at the lumbar spine, femoral neck, or total hip were classified as having osteoporosis. Patients with PSS were divided into pSS with OP and pSS without OP group according to whether they had osteoporosis. The study was reviewed and approved by the Ethics Committee of the Second Hospital of Shanxi Medical University (2025YX408).

Based on previous studies on common risk factors for osteoporosis, demographic characteristics and laboratory data were collected from all patients. These included gender, age, body mass index (BMI), symptoms such as dry mouth, dry eye, and severe tooth loss, smoking, drinking, menstrual status, arthralgia, as well as a series of laboratory parameters. The laboratory parameters comprised complete blood cell counts (white blood cells, red blood cells, hemoglobin, lymphocytes, monocytes, and neutrophils), erythrocyte sedimentation rate (ESR), fibrinogen level, activated partial thromboplastin time (APTT), thrombin time (TT), and immunoglobulins (IgA, IgG, IgM). Autoantibody profiles included antinuclear antibody (ANA), anti-extractable nuclear antigen (anti-ENA) antibodies, anti-Smith (anti-Sm) antibody, anti-SSA antibody, anti-SSB antibody, and anti-Ro52 antibody. Rheumatoid factor (RF) isotypes (RF-IgA, RF-IgG, RF-IgM), along with biochemical markers such as alanine aminotransferase (ALT), aspartate aminotransferase (AST), total protein (TP), albumin (ALB), Serum Alkaline Phosphataseurea(ALP), and calcium (Ca), were also measured. All laboratory tests were performed using automated hematology analyzers, indirect immunofluorescence assay, chemiluminescence immunoassay, and enzyme-linked immunosorbent assay in the Rheumatology and Immunology Laboratory of the Second Hospital of Shanxi Medical University. Not all variables were directly bone-specific; some were included as exploratory predictors because osteoporosis in pSS may be influenced by systemic inflammation and immune dysregulation. A summary of the collected clinical and laboratory data is provided in **Table 1**.

**Table 1:** Description of research factors.

Variables	Description	Types	Unit
Gender	Sex of the patient	Categorical	1 male 2 female
Age	Age of the patient	Continuous	year
BMI	Body Mass Index	Continuous	kg/m²
Dry mouth	Symptom of oral dryness	Categorical	0 No history of dry mouth 1 History of dry mouth
Dry eyes	Symptom of dry eyes	Categorical	0 No history of dry eyes 1 History of dry eyes
Smoking	Cigarette smoking status	Categorical	0 Non-smoker 1 Smoker
Drinking	Alcohol drinking status	Categorical	0 Non-drinker 1 Drinker
Menstrual status	Menstrual status	Categorical	0 Premenopausal 1 Postmenopausal NA = Not applicable (male)
Arthralgia	Presence of joint pain	Categorical	0 No history of arthralgia 1 History of arthralgia
Severe tooth loss	Tooth loss in chunks	Categorical	0 No history of lumpy tooth 1 History of lumpy tooth
WBC	White Blood Cell count	Continuous	10^9/L
RBC	Red Blood Cell count	Continuous	10^12/L
HB	Hemoglobin	Continuous	g/L
LYM	Lymphocyte count	Continuous	10^9/L
MON	Monocyte count	Continuous	10^9/L
NEU	Neutrophil count	Continuous	10^9/L
ESR	Erythrocyte Sedimentation Rate	Continuous	mm/H
Fibrinogen	Fibrinogen level	Continuous	g/L
APTT	Activated Partial Thromboplastin Time	Continuous	s
TT	Thrombin Time	Continuous	s
IgA	Immunoglobulin A	Continuous	g/L
IgG	Immunoglobulin G	Continuous	g/L
IgM	Immunoglobulin M	Continuous	g/L
ANA	Antinuclear Antibody	Categorical	0 Antinuclear antibody negative 1 Antinuclear antibody positive
Anti ENA	Anti-Extractable Nuclear Antigen antibody	Categorical	0 Anti-Extractable Nuclear Antigen antibody negative 1 Anti-Extractable Nuclear Antigen antibody positive
Anti Sm	Anti-Smith antibody	Categorical	0 Anti-Smith antibody negative 1 Anti-Smith antibody positive
Anti SSA	Anti-SSA antibody	Categorical	0 Anti-SSA antibody negative 1 Anti-SSA antibody positive
Anti SSB	Anti-SSB antibody	Categorical	0 Anti-SSB antibody negative 1 Anti-SSB antibody positive
Anti Ro52	Anti-Ro52 antibody	Categorical	0 Anti-Ro52 antibody negative 1 Anti-Ro52 antibody positive
RF-IgA	Rheumatoid Factor Immunoglobulin A	Categorical	0 Rheumatoid Factor Immunoglobulin A negative 1 Rheumatoid Factor Immunoglobulin A positive
RF-IgG	Rheumatoid Factor Immunoglobulin G	Categorical	0 Rheumatoid Factor Immunoglobulin G negative 1 Rheumatoid Factor Immunoglobulin G positive
RF-IgM	Rheumatoid Factor Immunoglobulin M	Categorical	0 Rheumatoid Factor Immunoglobulin M negative 1 Rheumatoid Factor Immunoglobulin M positive
ALT	Alanine Aminotransferase	Continuous	U/L
AST	Aspartate Aminotransferase	Continuous	U/L
TP	Total Protein	Continuous	g/L
ALB	Albumin	Continuous	g/L
ALP	Serum Alkaline Phosphatase	Continuous	U/L
Urea	Urea level	Continuous	mmol/L
Ca	Calcium	Continuous	mmol/L

### 2.2 Detection of lymphocyte subsets and cytokines by flow cytometry

Peripheral blood samples were collected from all subjects. To determine the absolute and relative counts of basic lymphocyte subsets, a mixture of fluorescein-labeled antibodies against lymphocyte surface markers was added to stain the cells. The samples were incubated for 15 to 20 minutes at room temperature in the dark. For T lymphocyte subset detection, anti-CD3, anti-CD8, anti-CD45, and anti-CD4 antibodies were used. For B lymphocytes and natural killer (NK) cells, anti-CD3, anti-CD16, anti-CD56, anti-CD45, and anti-CD19 antibodies were applied. After incubation, lysing solution was added to each tube, and the samples were incubated again in the dark for 15 minutes at room temperature. After lysis, the samples were washed with PBS and centrifuged to remove cell debris and unbound antibodies. Finally, the cells were resuspended in PBS and analyzed using a BD FACSCalibur flow cytometer.

To identify functional T cell subsets, peripheral blood mononuclear cells (PBMCs) were isolated and stimulated with phorbol myristate acetate (PMA), ionomycin, and protein transport inhibitors for 6 hours at 37 °C in a 5% CO_2_ atmosphere. The stimulated cells were then stained with surface markers, including CD3 and CD4, followed by fixation and membrane permeabilization. Subsequently, the cells were incubated with antibodies against intracellular cytokines such as interferon-gamma (IFN-γ), interleukin-4 (IL-4), and interleukin-17A (IL-17A). After washing, the samples were analyzed by flow cytometry. Regulatory T cell (Treg) detection was also performed using PBMC samples. After surface staining for CD3, CD4, and CD25, followed by red blood cell lysis, the cells were fixed and permeabilized. Subsequently, intracellular staining with anti-Foxp3 antibody was conducted.

Cytokine levels were measured from the collected culture supernatant using a cytometric bead array (CBA) kit. Microspheres with distinct fluorescence intensities were used to capture specific cytokines in the samples. After incubation with a phycoerythrin (PE)-labeled detection antibody mixture, the beads were washed, and their fluorescence was detected by flow cytometry. The fluorescent antibodies used in the experiments were purchased from BD Biosciences, and the CBA kits were obtained from Jiangxi Cell Gene Biotechnology Co., Ltd.

### 2.3 Characteristic factor screening

We adopted a combined feature-selection strategy based on least absolute shrinkage and selection operator (LASSO) regression, random forest (RF), and stepwise regression.

LASSO regression performs automatic variable selection by introducing an L1 penalty into the loss function, which shrinks the coefficients of less informative predictors toward zero. This method is particularly useful for handling multicollinearity and generating sparse solutions. RF was used to assess feature importance by calculating the mean decrease in impurity contributed by each variable across a large number of decision trees. This approach is capable of capturing complex nonlinear relationships and is relatively robust to outliers. Stepwise regression was also applied as a conventional regression-based variable reduction method, in which predictors are iteratively added or removed according to the trade-off between model fit and model complexity, yielding an optimized subset of variables.

These methods were selected because they capture different aspects of predictor relevance: LASSO is effective for coefficient shrinkage and collinearity handling, RF can detect nonlinear and complex associations, and stepwise regression provides a traditional regression-based simplification framework. To prioritize predictors with more robust cross-method support and a lower risk of method-specific selection bias, we retained variables that were consistently identified across methods. Finally, the intersection of the features selected by the three approaches was determined, and the resulting candidate predictors were further reviewed by clinical experts to assess their plausibility and practical interpretability.

### 2.4 Model construction and performance evaluation

A total of nine classification algorithms were developed: decision tree (DT), k-nearest neighbor (KNN), logistic regression, elastic net (eNet), random forest (RF), LightGBM, XGBoost, support vector machine (SVM), and multilayer perceptron (MLP). During model development, all models were evaluated using 5-fold cross-validation within the training set. For models requiring hyperparameter tuning, parameter optimization was performed using grid search, random search, or Bayesian optimization, depending on the characteristics of each algorithm. The optimal hyperparameter combination was selected according to the highest area under the receiver operating characteristic curve (AUC) achieved during cross-validation. Logistic regression was fitted using the glm engine, after identification of the optimal hyperparameters, each model was refitted on the full training set and subsequently evaluated in the internal test set. Model performance was assessed in terms of discrimination, classification performance, calibration, and potential clinical utility. Accuracy, AUC, and area under the precision-recall curve (PR-AUC) were used to evaluate discrimination and classification performance during resampling. For the binary classification task, the optimal classification threshold was determined in the training set using the Youden index derived from the ROC curve. This threshold was used to generate the confusion matrix for the training set and was subsequently fixed and applied to the internal test set for classification performance evaluation, including the calculation of sensitivity, specificity, positive predictive value (PPV), negative predictive value (NPV), and F1 score.

To further assess model robustness, generalizability, and calibration, the final candidate models underwent repeated cross-validation, bootstrap resampling, and calibration analysis. Repeated cross-validation was performed with 5 folds repeated 10 times, and bootstrap validation was conducted with 200 resamples. Accuracy, AUC, PR-AUC, and the Brier score were calculated during resampling to evaluate predictive performance and stability. Calibration performance was assessed in the internal test set. The Brier score was used to quantify the overall error between predicted probabilities and observed outcomes, with lower values indicating better calibration. Calibration intercept and calibration slope were further estimated to assess agreement between predicted and observed risks; a calibration intercept closer to 0 and a calibration slope closer to 1 indicated better calibration performance. In addition, calibration curves were plotted to visually evaluate the agreement between predicted and observed event probabilities. Decision curve analysis was also performed to assess the potential clinical net benefit of each model.

Finally, all candidate models were comprehensively compared in the internal test set across multiple dimensions, including discrimination, classification performance, calibration, and clinical utility. Evaluation metrics included AUC, accuracy, sensitivity, specificity, PPV, NPV, F1 score, calibration curves, and decision curve analysis. The model with the best overall performance and greatest potential for clinical application was selected as the final prediction model.

To provide deeper insight beyond conventional performance evaluation, the Shapley Additive Explanations (SHAP) framework was applied to interpret the final model. By calculating the average marginal contribution of each feature across all possible combinations, SHAP enabled attribution analysis of the model’s predictions. This approach quantitatively and consistently elucidated the key predictive factors, as well as the direction and magnitude of their impact on the model output. These interpretations enhance model transparency and credibility, while offering valuable insights for subsequent biological or clinical investigations.

### 2.5 Comparison with simplified clinical screening models

After developing and evaluating the candidate machine learning algorithms, the model with the best overall performance on the testing set was selected as the optimal model. To assess its potential added value over simplified clinical screening strategies, we further compared the selected optimal model with several low-dimensional and clinically interpretable screening approaches.

Specifically, three simplified models were constructed using readily available clinical variables: an Age-alone model, an Age-plus-Ca model, and a simplified screening model including Age, ESR, and Ca. These simplified models were fitted using logistic regression on the same training set and were evaluated on the same testing set as the optimal machine learning model. Model performance was compared using accuracy, sensitivity, specificity, PPV, NPV, F1 score, and AUC.

### 2.6 Statistical analysis

Prior to analysis, samples with more than 20% missing values and variables with more than 30% missingness were excluded. For the remaining data, missing values were handled using multiple imputation by chained equations (MICE) under the missing-at-random assumption. The imputation model included the outcome variable, all candidate predictors considered for feature selection and model development, and auxiliary variables associated with missingness where appropriate. Structural missingness due to non-applicability was not treated as missing data and was therefore not imputed. Multiple imputation was performed only for true missing values among applicable participants. Continuous variables were imputed using predictive mean matching, and binary variables using logistic regression. We generated 20 imputed datasets with 10 iterations each. Convergence was assessed by visual inspection of trace plots, and the plausibility of imputations was evaluated by comparing the distributions of observed and imputed values. For variable description and intergroup comparisons, categorical variables were presented as frequencies and percentages and analyzed using the chi-square test. Continuous variables were first assessed for normality using the Shapiro-Wilk test. Variables with a normal distribution were expressed as mean ± standard deviation and compared using the independent samples t-test. Non-normally distributed variables were expressed as median and compared using the Mann–Whitney U test. Given the number of variables compared between groups, *P* values from the univariate analyses were further adjusted for multiple testing using the Benjamini–Hochberg false discovery rate (FDR) method. Adjusted *P* values < 0.05 were considered statistically significant.

**Figure 1:**
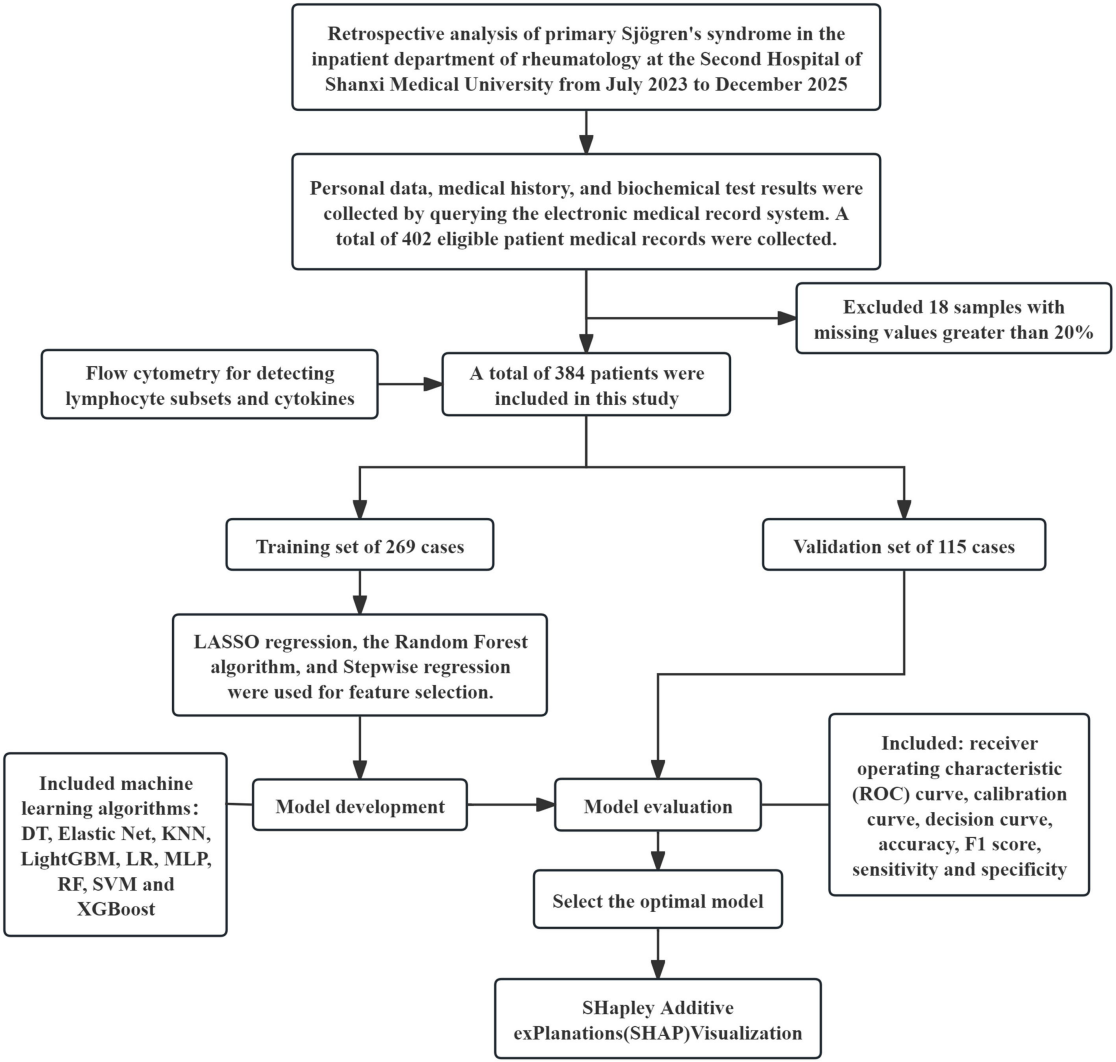
Research flowchart.

To identify key predictive features, a comprehensive screening approach was applied using Lasso regression, RF feature importance, and stepwise regression. Based on the selected features, nine machine learning models were constructed and compared: DT, KNN, logistic regression, elastic net, RF, LightGBM, XGBoost, SVM, and MLP. All machine learning analyses were conducted in R using the tidymodels framework with the corresponding model engines. After harmonizing variable data types, the dataset was randomly split into a training set and an internal test set at a ratio of 7:3 using stratified sampling based on the outcome variable. A fixed random seed was specified to ensure reproducibility. All data preprocessing, feature selection, model development, and hyperparameter tuning were performed exclusively in the training set, whereas the internal test set was reserved for final model validation. Data preprocessing was implemented using the recipe framework and dummy encoding of categorical variables. Model performance was evaluated on the independent test set from three perspectives: discriminative ability, calibration, and clinical utility. All statistical analyses were performed using SPSS version 27.0, R version 4.4.1, and Python version 3.7. Machine learning analyses were primarily implemented in R. The main R package used for model development and tuning was tidymodels (version 1.5.0). In addition, specific packages were used for particular algorithms, including randomForest (version 4.7) for RF, xgboost (version 3.2.1.1) for XGBoost, and bonsai (version 0.4.1) for LightGBM. The overall experimental workflow is illustrated in **Figure 1**.

## 3 Results

### 3.1 Comparison of patient characteristics between the pSS without OP group and the pSS with OP group

After data processing, 18 samples with more than 20% missing values were excluded. A total of 384 patients were ultimately included in this study. The de-identified raw data supporting the findings of this study are provided as **Supplementary Material 1**. Their baseline information, clinical characteristics, and laboratory results are summarized in **Table 2**. The cohort included 364 females (95%) and 20 males (5%), with a median age of 61.5 years (53,68). Based on clinical diagnosis, 102 patients (26.6%) were classified into the pSS with OP group. In this group, 96 patients (94%) were female and 6 (6%) were male, with a median age of 66 years (62,71). The remaining 282 patients were assigned to the pSS without OP group, comprising 268 females (95%) and 14 males (5%), with a median age of 60 years (51,68). No significant difference in gender distribution was found between the two groups. However, the age difference was statistically significant, with patients in the pSS with OP group being older than those in the pSS without OP group. In addition, the pSS with OP group had significantly higher levels of ESR, WBC, MON, Fibrinogen, Urea, RF-IgG, and RF-IgM, as well as a higher proportion of patients with postmenopausal. In contrast, the pSS without OP group showed significantly higher levels of TP, ALB, Ca, and TT compared to the pSS with OP group.

**Table 2:** Baseline information.

Variables	Total (n = 384)	pSS without OP (n = 282)	pSS with OP (n = 102)	Adjusted-p
Demographics				
Gender, n (%)				0.816
1	20 (5)	14 (5)	6 (6)	
2	364 (95)	268 (95)	96 (94)	
Age	61.5 (53, 68.25)	60 (51, 68)	66 (62, 71)	< 0.001***
BMI	22.93 (21.36, 24.73)	23.03 (21.31, 24.95)	22.68 (21.48, 24.22)	0.316
Clinical characteristics				
Dry mouth, n (%)				0.051
0	39 (10)	35 (12)	4 (4)	
1	345 (90)	247 (88)	98 (96)	
Dry eyes, n (%)				0.740
0	117 (30)	88 (31)	29 (28)	
1	267 (70)	194 (69)	73 (72)	
Severe tooth loss, n (%)				0.284
0	227 (59)	173 (61)	54 (53)	
1	157 (41)	109 (39)	48 (47)	
Smoking, n (%)				0.133
0	315 (88)	228 (87)	87 (94)	
1	41 (12)	35 (13)	6 (6)	
Drinking, n (%)				0.475
0	334 (94)	245 (93)	89 (96)	
1	22 (6)	18 (7)	4 (4)	
Menstrual status, n (%)				0.002**
0	87 (24)	77 (30)	10 (11)	
1	269 (76)	184 (70)	85 (89)	
Arthralgia, n (%)				0.498
0	199 (54)	147 (55)	52 (51)	
1	169 (46)	119 (45)	50 (49)	
Complete blood count				
WBC	5.54 (4.39, 7.6)	5.46 (4.08, 7.4)	6.14 (4.86, 8.18)	0.042*
RBC	4.16 (3.84, 4.48)	4.19 (3.86, 4.5)	4.12 (3.79, 4.4)	0.400
HB	126 (116.75, 135)	126 (117, 133.75)	129.5 (112.25, 135)	0.498
LYM	1.51 (1.04, 2.02)	1.5 (1.02, 2.02)	1.52 (1.1, 2.04)	0.816
MON	0.47 (0.31, 0.64)	0.42 (0.28, 0.6)	0.55 (0.42, 0.76)	<0.001***
NEU	3.26 (2.35, 5.05)	3.2 (2.26, 5.04)	3.63 (2.63, 5.04)	0.232
Immunoglobulin				
IgA	3.01 (2.22, 3.9)	3.01 (2.09, 3.94)	3 (2.45, 3.77)	0.284
IgG	15.47 (11.93, 19.59)	15.85 (11.86, 19.98)	14.5 (12.26, 17.5)	0.405
IgM	1.19 (0.84, 1.79)	1.19 (0.83, 1.92)	1.21 (0.86, 1.63)	0.977
Biochemical markers				
ALT	22.1 (15.4, 35.3)	21.4 (14.86, 35.2)	24.45 (17.4, 35.71)	0.894
AST	25 (20.15, 34.31)	24.2 (19.7, 35.6)	28.23 (21.93, 32.41)	0.316
TP	73.3 (68.81, 78.5)	74.1 (69.1, 79.65)	70.79 (67.15, 74.99)	0.002**
ALB	40.71 (37.25, 43.59)	41.7 (37.65, 44.05)	39.41 (36.7, 41.3)	0.003**
ALP	78 (63, 103)	76.5 (61, 99)	83 (65, 110)	0.178
Urea	5.4 (4, 6.6)	5.18 (3.9, 6.4)	5.92 (5.14, 7.45)	<0.001***
Ca	2.26 (2.18, 2.34)	2.29 (2.19, 2.37)	2.23 (2.17, 2.28)	0.002**
Laboratory characteristics				
RF-IgA, n (%)				0.740
0	173 (48)	129 (48)	44 (45)	
1	191 (52)	138 (52)	53 (55)	
RF-IgG, n (%)				0.003**
0	245 (67)	193 (72)	52 (54)	
1	119 (33)	74 (28)	45 (46)	
RF-IgM, n (%)				0.022*
0	245 (67)	193 (72)	52 (54)	
1	119 (33)	74 (28)	45 (46)	
ANA, n (%)				0.498
0	245 (67)	193 (72)	52 (54)	
1	119 (33)	74 (28)	45 (46)	
Anti ENA, n (%)				0.816
0	235 (64)	171 (63)	64 (66)	
1	134 (36)	101 (37)	33 (34)	
Anti Sm, n (%)				0.740
0	235 (64)	171 (63)	64 (66)	
1	134 (36)	101 (37)	33 (34)	
Anti SSA, n (%)				0.816
0	235 (64)	171 (63)	64 (66)	
1	134 (36)	101 (37)	33 (34)	
Anti SSB, n (%)				0.883
0	235 (64)	171 (63)	64 (66)	
1	134 (36)	101 (37)	33 (34)	
Anti Ro52, n (%)				0.316
0	235 (64)	171 (63)	64 (66)	
1	134 (36)	101 (37)	33 (34)	
SIL-2R	588.65 (369.36, 830.92)	553.62 (352.36, 808.73)	655.2 (448.61, 870.78)	0.185
ESR	22 (12, 38)	19 (12, 33)	32 (15, 45)	0.002**
Fibrinogen	3.12 (2.65, 3.56)	3.07 (2.61, 3.47)	3.24 (2.79, 3.74)	0.048*
APTT	28.62 (26.35, 31.1)	28.56 (25.95, 31.21)	28.67 (27.21, 30.43)	0.691
TT	16.29 (14.64, 17.78)	16.41 (15, 18.02)	15.47 (14.3, 16.86)	0.031*

*P<0.05, **P<0.01, ***P<0.001.

According to the flow cytometry analysis of lymphocyte subsets and cytokines (**Table 3**), the proportions of Th1, Th17, and Treg cells in the pSS with OP group were significantly lower than those in the pSS without OP group. In contrast, the NK/Treg ratio, absolute counts of IL-6, and NK cells, as well as the proportion of NK cells, were significantly higher in the pSS with OP group than in the pSS without OP group.

**Table 3:** Comparison of lymphocyte subsets and cytokines.

Variables	Total (n = 384)	pSS without OP (n = 282)	pSS with OP(n = 102)	Adjusted-p
Lymphocyte subsets				
Th1%	14.18 (9.25, 18.49)	14.55 (9.53, 19.81)	12.84 (8.64, 15.46)	0.008*
Th2%	1.16 (0.9, 1.52)	1.16 (0.9, 1.56)	1.15 (0.92, 1.4)	0.624
Th17%	1.44 (1.05, 1.95)	1.5 (1.13, 2)	1.31 (0.88, 1.81)	0.003**
Treg%	5.08 (4.16, 6.31)	5.28 (4.22, 6.52)	4.72 (3.82, 5.44)	0.023*
Th1	87.16 (47.4, 124.92)	88.14 (46.61, 127.39)	84.37 (49.18, 118.52)	0.498
Th2	7.21 (4.52, 9.76)	7.14 (4.42, 9.74)	7.33 (5.14, 9.67)	0.729
Th17	8.54 (6.07, 11.4)	8.9 (6.07, 12.01)	8.24 (6.34, 9.52)	0.284
Treg	31.35 (24, 39.62)	31.15 (22.82, 39.59)	31.9 (26.12, 40.22)	0.894
Th1/Th2	12.18 (8.35, 15.71)	12.56 (8.18, 16.79)	10.87 (8.69, 13.57)	0.071
Th17/Treg	0.28 (0.21, 0.39)	0.29 (0.21, 0.41)	0.27 (0.2, 0.35)	0.326
Th1/Treg	2.74 (1.65, 3.84)	2.7 (1.61, 3.87)	2.85 (1.81, 3.59)	0.904
Th2/Treg	0.23 (0.17, 0.31)	0.23 (0.16, 0.31)	0.24 (0.18, 0.3)	0.261
Bcell/Treg	7.03 (4.63, 10.12)	6.95 (4.35, 10.3)	7.23 (5.24, 9.7)	0.560
NKcelI/Treg	5.36 (2.86, 8.01)	5.1 (2.6, 7.71)	6.22 (4.44, 9.51)	0.003**
Total T	1063.52 (784.69, 1341.5)	1047.15 (745.77, 1299.7)	1104.97 (903.61, 1383.78)	0.130
Total T%	71.54 (65.98, 77.34)	72.07 (65.31, 77.47)	71.14 (67.85, 75.99)	0.816
Total B	213.1 (130.38, 303.42)	213.82 (121.51, 307.03)	212.38 (146.38, 270.79)	0.894
Total B%	14.96 (9.93, 20.19)	15.61 (9.98, 21.31)	14.34 (9.9, 16.87)	0.284
Th	584.72 (447.95, 820.51)	565.9 (425.36, 775.11)	641.02 (473.2, 850.2)	0.092
Th%	41.92 (34.8, 46.95)	41.75 (34.56, 46.94)	42.11 (36.16, 47.16)	0.603
Ts	381.3 (259.5, 522.94)	370.92 (244.56, 520.93)	400.78 (303.29, 530.01)	0.197
Ts%	26.72 (20.93, 32.83)	26.5 (20.88, 32.86)	26.86 (21.33, 32.01)	0.816
Th/Ts	1.59 (1.12, 2.16)	1.58 (1.12, 2.17)	1.6 (1.11, 2.15)	0.903
NK	168.04 (95.33, 244.12)	155.92 (81.1, 232.66)	188.72 (135.52, 270.12)	0.004**
NK%	12.04 (7.38, 15.83)	11.23 (6.53, 15.31)	12.57 (10.17, 17.66)	0.025*
TLC	1469.58 (1117.77, 1816.08)	1439.29 (1045.51, 1809.56)	1517.71 (1272.89, 1854.63)	0.167
TLC%	99.32 (99.08, 99.56)	99.31 (99.04, 99.56)	99.35 (99.16, 99.59)	0.284
Cytokines				
IL-2	1.73 (1.1, 2.44)	1.75 (1.04, 2.47)	1.64 (1.25, 2.13)	0.284
IL-4	2.41 (1.63, 3.1)	2.46 (1.63, 3.21)	2.19 (1.63, 2.87)	0.167
IL-6	5.76 (3.42, 10.43)	5.19 (3.29, 9.48)	6.91 (4.08, 15.04)	0.026*

*P<0.05, **P<0.01.

### 3.2 Screening of characteristic factors

**Figure 2:**
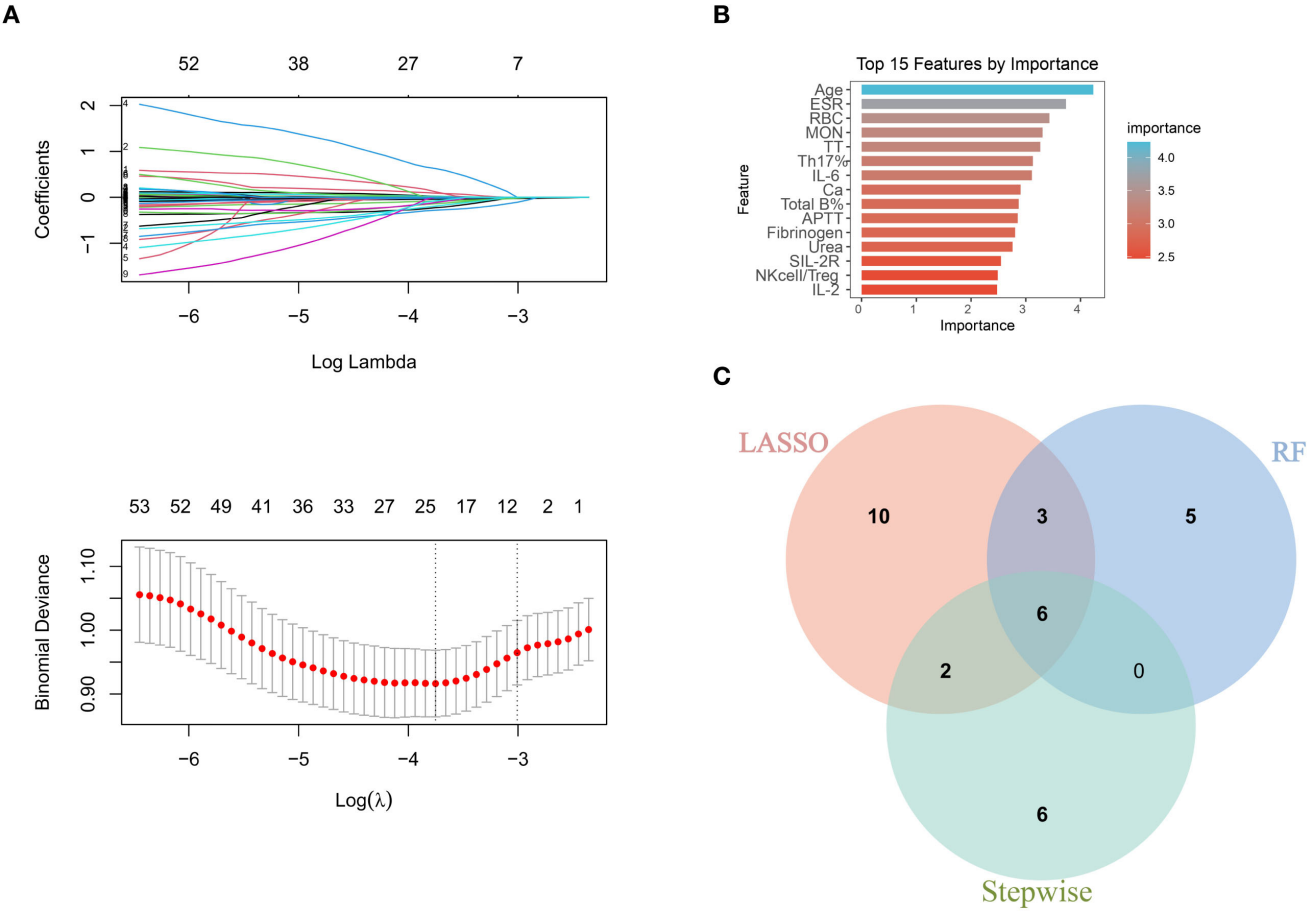
Screening of Characteristic Factors. **(A)** Result of LASSO. **(B)** Result of RF. **(C)** Venn diagram of three kinds of machine learning.

The patient cases were divided into a training set of 269 cases and an internal test set of 115 cases at a 7:3 ratio. In the training set, the LASSO algorithm was first used for feature screening. Based on variable importance, 22 key factors were retained (**Figure 2A**), including Age, ESR, WBC, MON, Fibrinogen, IL-4, IL-6, TP, Urea, Ca, TT, Th1%, Th17%, total T cells, total B%, Th cells, NK%, TLC%, dry mouth, RF-IgG, anti-Sm, and anti-Ro52. Additionally, the RF algorithm was applied for feature selection. The top 15 variables were identified based on importance scores (**Figure 2B**), including Age, ESR, RBC, MON, TT, Th17%, IL-6, Ca, total B%, APTT, Fibrinogen, Urea, SIL-2R, NK/Treg ratio, and IL-2. Stepwise regression was also performed, which identified Age, Th cells, RF-IgG, NK%, ESR, Urea, MON, Ts%, Th/Ts ratio, Th1/Treg ratio, IL-4, Ca, Hb, and Fibrinogen as candidate features (**Table 4**). Finally, by integrating the intersecting results from the three machine learning methods and incorporating clinical expert opinions, the following variables were selected for final modeling: Age, ESR, Urea, MON, Ca, and Fibrinogen (**Figure 2C**). These characteristics showed statistical significance.

**Table 4:** Results of characteristic factor screening.

Method type	Characteristic factors
LASSO	Age, ESR, WBC, MON, Fibrinogen, IL-4, IL-6, TP, Urea, Ca, TT, Th1%, Th17%, total T cells, total B%, Th cells, NK%, TLC%, dry mouth, RF-IgG, anti-Sm, anti-Ro52
RF	Age, ESR, RBC, MON, TT, Th17%, IL-6, Ca, total B%, APTT, Fibrinogen, Urea, SIL-2R, NK/Treg ratio, IL-2
Stepwise regression	Age, Th cells, RF-IgG, NK%, ESR, Urea, MON, Ts%, Th/Ts ratio, Th1/Treg ratio, IL-4, Ca, Hb, Fibrinogen
Intersecting results	Age, ESR, Urea, MON, Ca, Fibrinogen

### 3.3 Prediction model construction and validation

**Figure 3:**
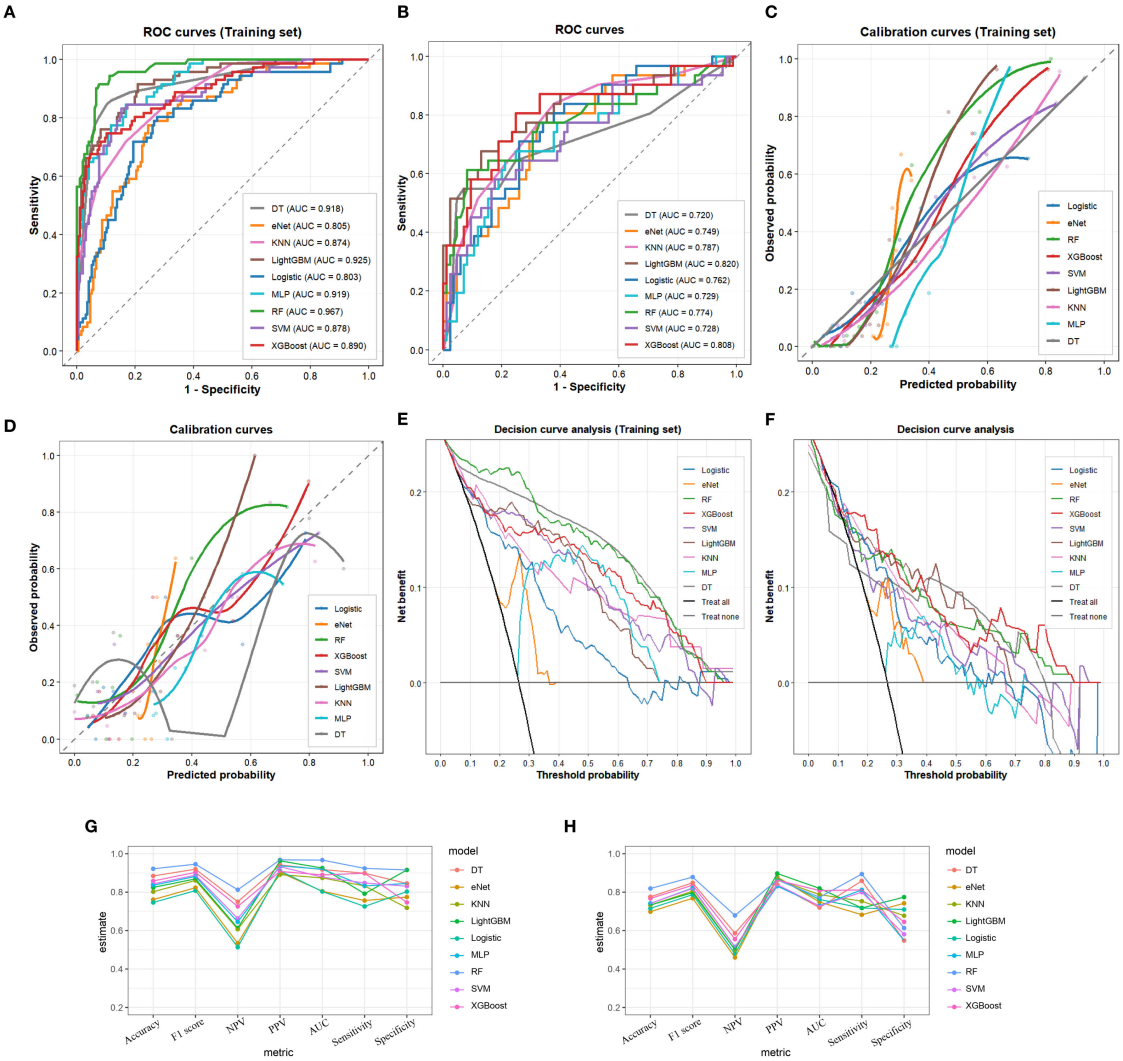
Validation of nine prediction models. **(A)** ROC curves in the training set. **(B)** ROC curves in the internal test set. **(C)** Calibration curves in the training set. **(D)** Calibration curves in the internal test set. **(E)** Decision curves in the training set. **(F)** Decision curves in the internal test set. **(G)** Parallel line graph of model performance in the training set. **(H)** Parallel line graph of model performance in the internal test set.

Nine machine learning models were developed and validated. In the training set, the AUC values were as follows: RF: 0.967 (95% CI: 0.936–0.998); LightGBM: 0.925 (95% CI: 0.883–0.967); MLP: 0.919 (95% CI: 0.874–0.963); DT: 0.918 (95% CI: 0.873–0.963); XGBoost: 0.890 (95% CI: 0.837–0.943); SVM: 0.878 (95% CI: 0.821–0.934); KNN: 0.874 (95% CI: 0.817–0.931); eNet: 0.805 (95% CI: 0.730–0.880); and Logistic: 0.803 (95% CI: 0.728–0.878) (**Figure 3A**). In the internal test set, the corresponding AUCs were: LightGBM: 0.820 (95% CI: 0.723–0.917); XGBoost: 0.808 (95% CI: 0.709–0.907); KNN: 0.787 (95% CI: 0.684–0.890); RF: 0.774 (95% CI: 0.669–0.879); Logistic: 0.762 (95% CI: 0.655–0.869); eNet: 0.749 (95% CI: 0.641–0.857); MLP: 0.730 (95% CI: 0.619–0.841); SVM: 0.728 (95% CI: 0.617–0.839); and DT: 0.720 (95% CI: 0.608–0.832) (**Figure 3B**).

We found substantial discrepancies between the training and test AUCs for DT, RF, and MLP, suggesting varying degrees of overfitting in all three models. To further evaluate their generalizability, we comprehensively assessed these models using repeated cross-validation, bootstrap validation, and calibration analysis.

Internal validation showed that the RF model achieved an AUC of 0.801 and a Brier score of 0.143 in repeated cross-validation, which were close to the corresponding bootstrap results (AUC = 0.783, Brier score = 0.149), indicating good stability and generalizability. Similarly, the MLP model yielded an AUC of 0.712 and a Brier score of 0.374 in repeated cross-validation, compared with an AUC of 0.701 and a Brier score of 0.381 in bootstrap validation, suggesting relatively consistent internal validation results, although its overall predictive performance was modest. In contrast, the DT model showed an AUC of 0.714 in repeated cross-validation, which declined to 0.667 in bootstrap validation, indicating limited stability and generalizability.

Calibration analysis further demonstrated that the RF model had a Brier score of 0.148, a calibration intercept of 0.213, and a calibration slope of 0.812 on the test set, indicating generally good calibration, although with slight risk underestimation and somewhat over-extreme predicted probabilities. The MLP model had a Brier score of 0.184, a calibration intercept of -0.604, and a calibration slope of 1.316, suggesting a tendency to overestimate the risk of positive events and overall moderate calibration performance. Although the DT model achieved a test-set Brier score of 0.157, its calibration intercept was -0.839 and its calibration slope was only 0.035, indicating marked risk overestimation and poor calibration.

Overall, the RF model outperformed DT and MLP in terms of discrimination, stability, and calibration. The DT model showed more pronounced overfitting and insufficient generalizability, whereas the MLP model was relatively stable but demonstrated only modest overall predictive and calibration performance.

During model selection, although the AUC analysis indicated that the RF model exhibited the highest performance, the calibration curves (**Figures 3C, D**) revealed that XGBoost, Logistic regression, and SVM had overall trajectories closer to the diagonal. These models showed smaller deviations and smoother curves, indicating optimal stability and predictive consistency. In contrast, the RF model demonstrated acceptable calibration performance in the training set, but its curve was significantly above the diagonal in the internal test set. This finding suggests a systematic tendency to underestimate event probabilities. In the decision curve analysis (**Figures 3E, F**), XGBoost, RF, and LightGBM exhibited good clinical applicability. To comprehensively evaluate model performance, this study further employed a parallel line plot system to compare multiple metrics, including accuracy, F1 score, positive predictive value (PPV), negative predictive value (NPV), AUC, sensitivity, and specificity (**Figures 3G, H**). In the training set, XGBoost outperformed LightGBM in accuracy (0.86 vs 0.82), sensitivity (0.90 vs 0.79), NPV (0.73 vs 0.61), and F1 score (0.90 vs 0.87). This advantage remained in the internal test set, where XGBoost achieved higher accuracy (0.77 vs 0.73), sensitivity (0.81 vs 0.72), NPV (0.56 vs 0.50), and F1 score (0.84 vs 0.80). Based on the comprehensive comparative analysis of these multidimensional performance indicators, XGBoost demonstrated optimal and robust discriminative ability in both the training and internal test sets, with the highest estimation accuracy. Therefore, XGBoost was ultimately selected as the optimal predictive model for this study.

### 3.4 XGBoost model description

**Figure 4:**
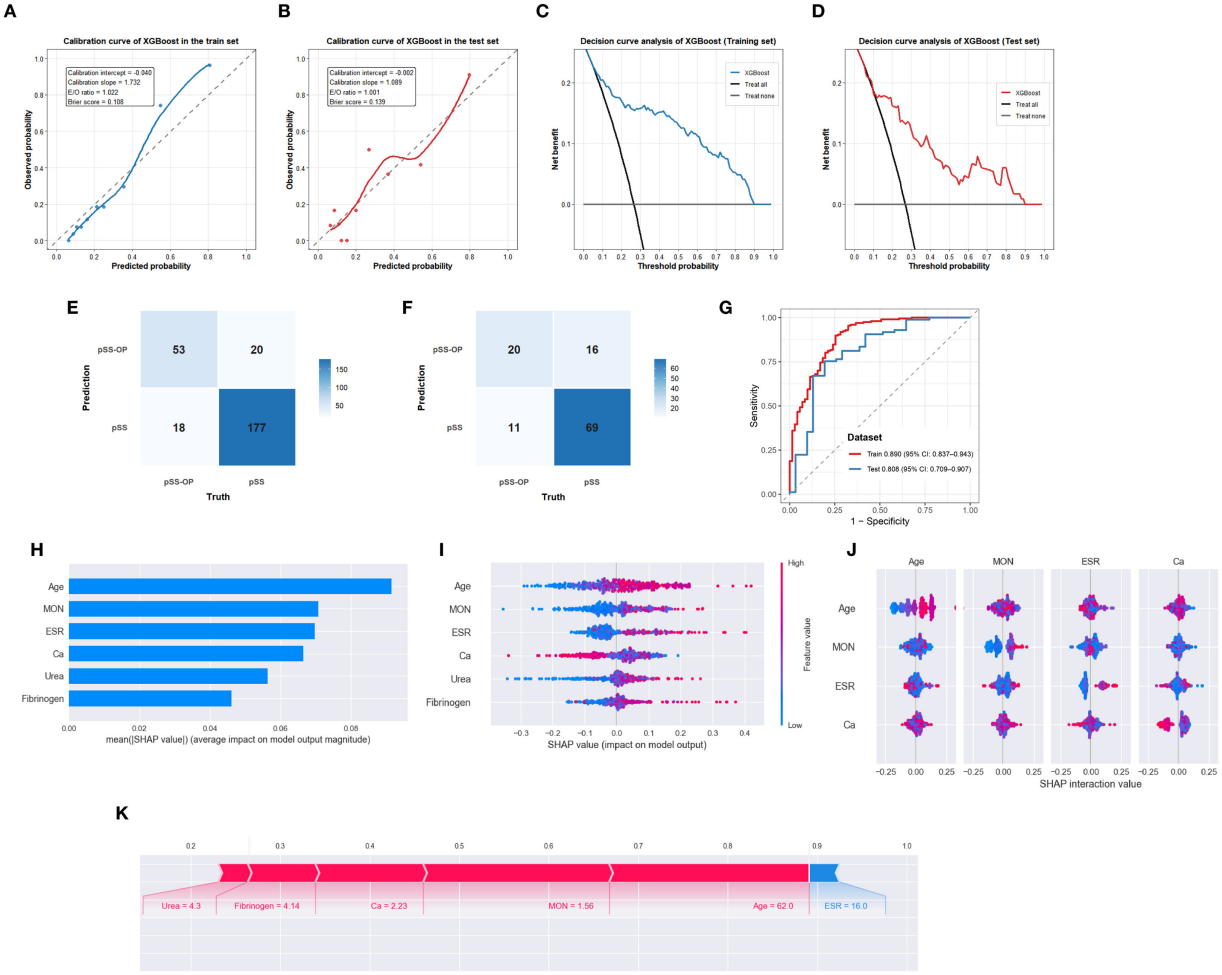
Comprehensive evaluation of the XGBoost model. **(A)** Calibration curve for the training set. **(B)** Calibration curve for the internal test set. **(C)** Decision curve for the training set. **(D)** Decision curve for the internal test set. **(E)** Confusion matrix for the training set. **(F)** Confusion matrix for the internal test set. **(G)** ROC curve of the model. **(H)** Feature importance ranking based on mean |SHAP|. **(I)** SHAP summary bee swarm plot. **(J)** Feature interaction plot. **(K)** SHAP force plot for a single-sample explanation.

This study systematically evaluated the performance of the XGBoost machine learning model on both the training set and the internal test set, aiming to assess its effectiveness and application potential. Analysis of the relevant graphical results indicated that the model performed well on both datasets. The comparison of predicted versus actual probabilities (**Figures 4A, B**) demonstrated a high degree of fit, with predicted values closely approximating actual outcomes, particularly in the high-probability interval. The net benefit curves (**Figures 4C, D**) showed that the model outperformed both the “treat all” and “treat none” strategies within the low-threshold range, thereby improving diagnostic net benefit. The optimal classification threshold determined by the Youden index from the ROC curve of the training set was 0.378. The confusion matrices (**Figures 4E, F**) further reflected the model’s stable classification ability. In the training set, the model correctly identified 177 true negatives and 53 true positives. In the internal test set, it identified 69 true negatives and 20 true positives. The ROC curves and corresponding AUC values also supported the model’s strong performance (**Figure 4G**). The AUC was 0.890 (95% CI: 0.837–0.943) in the training set and 0.808 (95% CI: 0.709–0.907) in the internal test set. Although the latter showed a slight decrease, it still indicated reliable discriminative ability. In summary, the XGBoost model demonstrated good predictive stability and diagnostic benefit in both the training and validation phases, highlighting its potential for practical application.

SHAP analysis was performed to explore the contribution and influence of each feature on the model’s predictions. The feature importance ranking, based on the mean absolute SHAP value (**Figure 4H**), showed that age had the greatest impact on the model output, exhibiting a strong positive effect on the prediction results. This was followed by MON, ESR, and Ca, which also demonstrated relatively important influences. In contrast, Urea and Fibrinogen had comparatively smaller effects. These findings highlight the key role of age in this prediction task, which may be related to its general influence on health status. The single-feature SHAP summary plot (**Figure 4I**) further supported these results. It illustrated the distribution of SHAP values for features such as Age, MON, and ESR across high and low value ranges, indicating that higher age values were strongly and positively associated with the model’s predictions. The feature interaction plot (**Figure 4J**) quantified and visualized the interaction effects between age and other features, including MON and ESR. This revealed more complex nonlinear relationships among these variables. Finally, a SHAP force plot for a specific case (**Figure 4K**) provided an intuitive visualization of how each feature contributed to the model’s decision for that individual. For instance, Age and ESR had notable impacts: increased age exerted a strong positive driving effect on the prediction, while ESR showed a negative influence to a certain extent.

A web-based calculator was developed using Shiny in R to facilitate individualized risk estimation based on the final XGBoost model. The calculator accepts user-entered predictor values, applies the same preprocessing pipeline as used in model development, and returns the predicted probability of the outcome. The calculator is available at: [https://dongjingqi.shinyapps.io/xgboost-r/].

To facilitate reproducibility and future validation, the implementation details of the final XGBoost model, including missing-data handling, data preprocessing, hyperparameter tuning, final model fitting, and performance evaluation, are provided in the **Supplementary Material 2**.

### 3.5 Comparison with simplified clinical screening models

**Figure 5:**
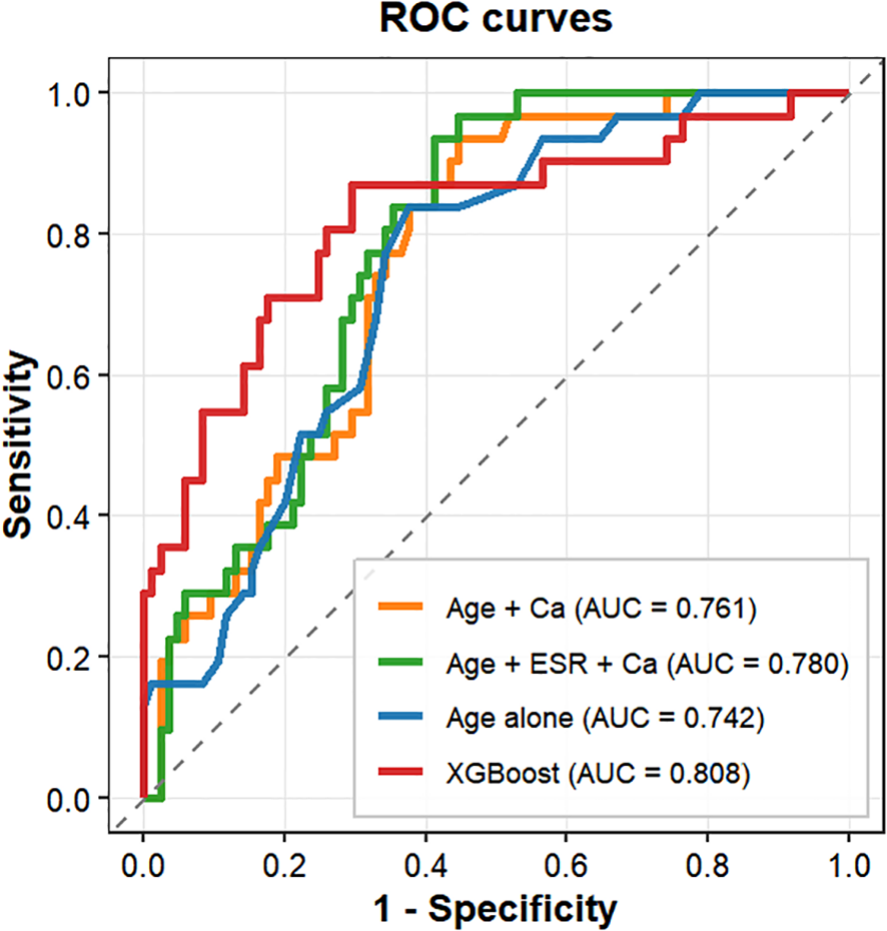
ROC curves in the internal test set of the XGBoost model and the simplified clinical screening models.

To further evaluate the clinical relevance of the XGBoost model, we compared it with several simplified screening approaches. On the testing set, XGBoost achieved the best overall performance, with the highest accuracy (0.767), F1 score (0.836), and AUC (0.808)(**Figure 5**). Its sensitivity (0.812) remained high and was comparable to that of the Age-alone and Age-plus-Ca models (both 0.839), while exceeding that of the simplified screening model (0.774). In terms of specificity, XGBoost (0.645) performed better than the Age-alone (0.553) and Age-plus-Ca (0.588) models, although it was slightly lower than the simplified screening model (0.671). Overall, these findings suggest that XGBoost offers improved predictive performance over simpler clinical screening approaches without a substantial loss of sensitivity.

## 4 Discussion

In this study, we developed and internally validated machine learning models to predict osteoporosis in patients with pSS. Among the nine candidate algorithms, XGBoost showed the best overall performance and was therefore selected as the final model. SHAP analysis further improved interpretability by identifying the major contributors to model predictions. To our knowledge, this study is among the first to combine machine learning–based prediction and explainable analysis in the specific clinical context of osteoporosis risk in pSS.

In this study, nine machine learning models were constructed and their performance was validated. The ROC curve was used to evaluate model discrimination, defined as the ability to distinguish between different categories. By plotting the true positive rate against the false positive rate, the AUC was calculated to quantify discriminative ability. An AUC value closer to 1 indicates stronger discrimination (27). The prediction models in this study demonstrated good discriminative ability. In the training set, the mean AUC was 0.89 (range: 0.80-0.97), and in the internal test set, the mean AUC was 0.76 (range: 0.72-0.82), both reaching an acceptable level. However, the performance gap between the training and internal test sets suggests that the models may exhibit a certain degree of overfitting. Therefore, to comprehensively evaluate their clinical utility, we went beyond discrimination alone and performed an integrated assessment incorporating calibration curves, decision curve analysis, repeated cross-validation, bootstrap validation, and calibration metrics.

Calibration curves were used to assess the consistency between predicted probabilities and actual observed probabilities. Ideally, the plotted curve should lie close to the diagonal line (28). Decision curve analysis was used to evaluate the clinical utility of the models. By comparing the strategies of using the model, intervening in all patients, or intervening in none, this approach helps weigh the clinical consequences of true positives and false positives (29). Comparative analysis of the calibration curves revealed the following: XGBoost, Logistic regression, and SVM exhibited smooth curves close to the ideal diagonal in both datasets, indicating the best probability consistency. Decision curve analysis of the training and internal test sets showed that RF, XGBoost, and LightGBM performed consistently and conservatively across both datasets. Their net benefit curves remained above the “treat none” strategy within the low-to-moderate threshold range of 5%–40%, suggesting potential clinical utility for risk-based screening. This threshold range is clinically relevant because the model is intended to identify patients with pSS who may benefit from further osteoporosis evaluation, particularly DXA screening, which is typically considered at low-to-moderate estimated risk. Lower thresholds may be more suitable when minimizing missed cases is prioritized, whereas higher thresholds may be preferable in settings with limited screening resources. Although DT, RF, and MLP all showed some degree of overfitting, RF demonstrated high consistency across repeated cross-validation and bootstrap validation, indicating better stability and generalizability than DT and MLP. Calibration analysis further showed that RF achieved the closest agreement between predicted and observed risks, despite slight risk underestimation and mildly overconfiden probability estimates. In contrast, DT exhibited poorer stability and clear overfitting with limited generalizability, while MLP showed relatively stable but only modest discriminative ability and suboptimal calibration. This multidimensional evaluation enabled a more rigorous and comprehensive appraisal of the true performance of the models.

The SHAP analysis showed that age was the most influential predictor, which is consistent with the well-established association between aging and bone loss (30). In addition, ESR and MON contributed substantially to the prediction, supporting the relevance of systemic inflammation in osteoporosis risk among patients with pSS (31, 32). In the context of rheumatic autoimmune diseases, accumulating evidence links chronic systemic inflammation to bone loss (33, 34). Serum calcium showed a negative association with osteoporosis risk in the model. Studies have shown that low calcium levels can indirectly promote bone resorption by enhancing parathyroid hormone (PTH) secretion or activating other compensatory mechanisms, thereby increasing osteoporosis risk (30). In contrast, Urea and Fibrinogen contributed relatively little to the model output. This may reflect their role as indirect markers of renal function or systemic inflammation rather than direct drivers of bone metabolism.

We further compared XGBoost with several simplified and clinically interpretable screening approaches. The results showed that XGBoost provided the best overall predictive performance, achieving the highest accuracy, F1 score, and AUC among the evaluated models. Notably, its sensitivity remained high and was broadly comparable to that of the simpler Age-based models, suggesting that the improved overall discrimination of XGBoost was not obtained at the expense of a major reduction in case detection. Although its specificity was slightly lower than that of the simplified clinical screening model, it remained higher than that of the Age-alone and Age-plus-Ca models. Taken together, these findings indicate that XGBoost may provide added value beyond simplified clinical screening approaches by improving overall classification performance while preserving clinically acceptable sensitivity.

Based on the comparison of clinical characteristics between the two groups and previous studies linking immune inflammation to bone metabolism, our findings suggest that patients with pSS and osteoporosis may have a greater systemic inflammatory burden. In this cohort, the osteoporosis group showed higher levels of ESR, WBC, MON, Fibrinogen, and IL-6, supporting a potential link between inflammation and abnormal bone metabolism. Previous studies have shown that IL-6 is involved in regulation of the RANKL/RANK/OPG axis and osteoclast differentiation (35), whereas ESR, WBC, and Fibrinogen may more broadly reflect systemic inflammatory activity associated with altered bone metabolism, although they do not directly prove inflammation-driven bone loss. Higher levels of RF IgG and RF IgM were also observed in the osteoporosis group, suggesting a more prominent B cell-mediated autoimmune phenotype. In addition to immune-related factors, nutritional and metabolic status may also be relevant. Lower TP, ALB, and serum calcium, together with higher urea levels, may reflect chronic inflammation-related nutritional depletion, impaired mineral homeostasis, and metabolic disturbance, respectively. Previous evidence has also suggested that low albumin is associated with reduced BMD and increased fracture risk (36). However, these associations still require confirmation in longitudinal studies.

The assessment of lymphocyte subsets in this study was based on the potential link between immune dysregulation and bone metabolism. The osteoporosis group showed a lower proportion of Treg cells and a higher NK/Treg ratio, suggesting a possible imbalance between immune regulation and innate immune activation. Previous studies have indicated that Treg cells may inhibit osteoclastogenesis through IL-4, IL-10, TGF-β, and CTLA-4-related pathways (37), whereas increased NK cells may reflect enhanced innate immune activation (38). Therefore, these findings are more supportive of an association with osteoporosis status than of a direct pathogenic role. The findings for T helper cell subsets were more complex. Although Th17 cells and related cytokines are generally considered to promote osteoclastogenesis (39), we observed lower peripheral Th17 and Th1 proportions in the osteoporosis group. They may reflect redistribution of these cells to inflamed tissues or bone marrow, treatment effects, or immune dynamics related to disease stage (40). In addition, Th1-related cytokines such as IFN-γ may exert dual effects on osteoclastogenesis depending on the inflammatory and hormonal context (41). Taken together, the effects of Th17 and Th1 cells on bone may be dual and dynamic, and their roles should be interpreted cautiously. From this perspective, osteoporosis in patients with pSS may not be merely an age-related comorbidity but may also be associated with the underlying autoimmune and inflammatory milieu.

Overall, these findings suggest that systemic inflammation, immune dysregulation, and nutritional-metabolic abnormalities may jointly contribute to osteoporosis in patients with pSS. Notably, although a broad range of immune cells and cytokines was assessed, most were not retained in the final model. This suggests that the final model should be interpreted as an integrated prediction tool based on demographic, biochemical, and inflammation-related features, rather than as a purely immune biomarker-based model.

From a clinical standpoint, the model is intended primarily as a risk stratification tool rather than a direct basis for treatment decisions. In practice, patients identified as high risk could be prioritized for further osteoporosis evaluation, such as DXA screening, laboratory assessment of bone-related metabolic factors, and multidisciplinary review when appropriate. This may be particularly relevant in pSS, where osteoporosis can remain underrecognized until substantial bone loss or fracture occurs. At the same time, the current model is not sufficient to define a universal intervention threshold for routine clinical implementation. Because this was a retrospective single-center study without external validation or threshold optimization, the predicted probabilities should be interpreted as supportive information for clinical assessment rather than as stand-alone decision criteria. Future studies are needed to determine clinically actionable cutoff values under different resource settings and screening objectives.

Several limitations should be acknowledged. First, although the study included 384 patients and 102 osteoporosis events, the effective sample size remained limited for the development and comparison of multiple machine learning algorithms, especially given the relatively large candidate predictor pool. Second, only internal validation was performed; therefore, the apparent advantage of XGBoost over the other models should be considered exploratory until confirmed in external datasets. Third, this was a single-center cohort from Shanxi, with a predominantly female population, which may limit generalizability to other regions, ethnic groups, healthcare settings, or male patients. In addition, some clinically relevant factors could not be adequately incorporated. Vitamin D and parathyroid hormone data had substantial missingness and were therefore excluded. Detailed glucocorticoid exposure, standardized pSS activity measures, and some disease-severity indicators were also unavailable. These limitations may have introduced residual confounding and restrict mechanistic interpretation. Finally, because detailed information on prior or ongoing anti-osteoporosis therapy was not comprehensively available, the potential confounding effects of these treatments on serum calcium levels, BMD, and the observed associations could not be fully excluded.

Future work should focus on external validation in larger, multicenter cohorts with broader demographic and clinical heterogeneity. Prospective studies are also needed to evaluate model transportability, recalibration, and clinical usefulness in real-world screening pathways. In addition, future datasets should include more complete and standardized information on disease activity, treatment exposure, bone turnover markers, vitamin D-related metabolism, and longitudinal bone mineral density outcomes. Such efforts would not only strengthen predictive performance but also help clarify the biological relationship between immune dysregulation and osteoporosis in pSS.

In conclusion, we identified several clinical and laboratory differences between pSS patients with and without osteoporosis and developed machine learning models for osteoporosis risk prediction. Among them, XGBoost showed the best overall performance in internal validation. SHAP analysis highlighted the relative contributions of Age, ESR, Urea, MON, Ca, and Fibrinogen, providing an interpretable view of model predictions. These findings offer a useful basis for early risk assessment and provide a foundation for future validation and refinement of individualized bone health management in pSS.

## Data Availability

The original contributions presented in the study are included in the article/**Supplementary Material**. Further inquiries can be directed to the corresponding author.
