# Supplementary material for: Immune-inflammatory and metabolic signatures for osteoporosis risk stratification in primary Sjögren’s syndrome: development and internal validation of an interpretable machine-learning model
Source: Front Immunol. 2026 Jul 10;17:1812285. doi: 10.3389/fimmu.2026.1812285 (PMC13396252; doi:10.3389/fimmu.2026.1812285)
Supplement: Supplementary file 1 [file DataSheet1.pdf]

1. Software environment:
R version: R 4.4.1
Packages:
tidymodels
mice
naniar
xgboost
skimr
dplyr
ggplot2
broom
Random seeds:
dataset split: 999
hyperparameter tuning: 666
repeated cross-validation: 2024
bootstrap: 2024

2. Data preparation and variable coding

All analyses were conducted in R. The binary outcome was coded as 0 and 1, with 1 representing the positive class. Variable types were harmonized before analysis, and categorical predictors were converted to factor variables where appropriate.

3. Screening for excessive missingness

To reduce the impact of excessive missingness, variables and samples were screened before imputation. Candidate predictors with a missing rate greater than 30% were excluded from model construction. Samples with a missing rate greater than 20% across candidate predictors were also excluded. Samples with missing outcome values were removed before analysis.

4. Train-test split

After missingness-based exclusion, the dataset was randomly divided into a training set (70%) and an internal test set (30%) using stratified sampling according to the outcome. Fixed random seeds were used to ensure reproducibility. All subsequent model-building procedures were conducted using the training set, whereas the internal test set was only used for final model validation.

5. Multiple imputation by chained equations

Remaining missing values were handled using multiple imputation by chained equations (MICE). To avoid information leakage, imputation was performed separately in the training set and the internal test set. The imputation model included the outcome variable, all candidate predictors retained after missingness-based exclusion, and, where applicable, auxiliary variables considered informative for the missing-data mechanism. The outcome variable was used as a predictor in the imputation models for other variables but was not itself imputed.

The number of imputations was set to m = [5], and the maximum number of iterations was set to maxit = [10]. Imputation methods were assigned according to variable type: predictive mean matching for continuous variables, logistic regression for binary variables, multinomial logistic regression for unordered categorical variables, and proportional odds logistic regression for ordered categorical variables. After imputation, a completed dataset was generated for downstream model construction. Convergence of the imputation process was assessed using trace plots, and the plausibility of imputed values was evaluated by comparing the distributions of imputed and observed data.

6. Data preprocessing after imputation

After imputation, preprocessing was performed using the recipe framework. Categorical predictors were transformed into dummy variables based on the preprocessing recipe derived from the imputed training data, and the same transformation structure was then applied to the imputed test data. Continuous variables were entered into the model on their original scales.

7. XGBoost model specification

The prediction model was developed using extreme gradient boosting (XGBoost) for binary classification. The model was implemented through the boost_tree() interface with the xgboost engine. The total number of trees was fixed at 1000, and early stopping was enabled with a stopping criterion of 25 rounds. An internal validation fraction of 0.2 was used during model fitting.

8. Hyperparameter tuning and model selection

Hyperparameter tuning was performed in the training set using stratified 5-fold cross-validation. The tuned hyperparameters included the number of predictors randomly sampled at each split (mtry), the minimum number of observations in terminal nodes (min_n), the maximum tree depth (tree_depth), the learning rate (learn_rate), the minimum loss reduction required for further partitioning (loss_reduction), and the subsampling proportion (sample_size). Candidate hyperparameter combinations were generated using random search. Model performance during tuning was summarized using accuracy, ROC-AUC, precision-recall AUC (PR-AUC), and Brier score, and the final hyperparameter combination was selected according to the highest ROC-AUC.

9. Final model fitting and prediction

After hyperparameter optimization, the final XGBoost workflow was refitted using the full imputed training dataset. The final model generated predicted probabilities for the positive class. For binary classification, the optimal probability threshold was determined in the training set using the Youden index from the ROC curve and subsequently applied to the internal test set.

10. Model performance evaluation

Model performance was evaluated in both the training set and the internal test set. Discrimination was assessed using ROC-AUC and PR-AUC. Classification performance was summarized using accuracy and confusion matrices. Probabilistic prediction error was evaluated using the Brier score. In addition, repeated stratified 5-fold cross-validation and bootstrap resampling were performed in the training set to further assess model robustness.

11. Calibration assessment

Calibration was evaluated in the internal test set using the Brier score, calibration intercept, and calibration slope. Calibration intercept was estimated by fitting a logistic regression model with the predicted logit included as an offset term, and calibration slope was estimated by regressing the observed outcome on the predicted logit. A grouped calibration plot based on deciles of predicted risk was also constructed.

12. Model interpretability

To improve interpretability, feature importance was quantified using XGBoost importance statistics, and SHAP-based visualizations were generated to show the contribution of major predictors to model predictions.
